# The effectiveness of naltrexone combined with current smoking cessation medication to attenuate post smoking cessation weight gain: a literature review

**DOI:** 10.1186/s40545-017-0109-7

**Published:** 2017-07-11

**Authors:** Raewyn Rees, Ali Seyfoddin

**Affiliations:** 1 School of Interprofessional Health Studies, Faculty of Health and Environmental Sciences, Auckland University of Technology, Auckland, New Zealand; 2 Drug Delivery Research Group, School of Science, Faculty of Health and Environmental Sciences, Auckland University of Technology, Auckland, New Zealand

**Keywords:** Naltrexone, Smoking cessation, Weight gain

## Abstract

**Background:**

Smoking is the number one cause of preventable morbidity and mortality globally and although many countries have invested heavily in smoking cessation programs, 21% of the global population still smoke. Post cessation weight gain has been identified as a barrier to attempting cessation and is implicated in the high rates of relapse. Naltrexone has been touted as a possible solution to address post smoking cessation weight gain.

**Results:**

The results from seven original studies assessing the effectiveness of naltrexone in combination with existing smoking cessation medications to attenuate post smoking cessation weight gain were obtained and critically reviewed. Five returned positive results and two returned results that were statistically insignificant. The positive results were seen more often in those identified as more likely to exhibit hedonic eating behaviour for example women and participants who were categorised as overweight or obese.

**Conclusion:**

The evidence suggests further investigation in to a combination of naltrexone and approved smoking cessation medications is warranted and could provide a solution to attenuate post smoking cessation weight gain especially in women and those classified as overweight or obese. This may provide the tool required to remove a perceived barrier to smoking cessation and improve global statistics.

## Background

Tobacco smoking is the leading cause of preventable morbidity and mortality globally and is causally linked to over five million deaths per year [1, 2]. There is overwhelming evidence that indicates it is the primary cause of nine different cancers and it is also implicated as a risk factor for stroke, cardiovascular disease, and numerous respiratory disorders [3]. Smoking affects every organ in the body and the financial burden for already stretched healthcare systems is crippling. With guidance from the World Health Organisation’s framework convention for tobacco control, many countries have invested in smoking cessation programs to try and reduce the scourge [4, 5]. New Zealand is regarded as a leader in tobacco control with bold initiatives including Smokefree 2025 (less than 5 % of the population smoking by 2025) paving the way [6]. However, although smoking rates have been steadily reducing there are still sectors within society that are overrepresented in the statistics. For example, according to the Ministry of Health, New Zealand Health Survey 2014/2015, 42 % of Maori women reported being current regular smokers, in comparison with 17 % of the total population [7]. Under article three of the Treaty of Waitangi, New Zealand’s founding document, the Crown has an obligation to ensure Maori are afforded oritetanga (equity) with non-Maori so action is needed [8]. Initiatives to improve smoking cessation statistics have been employed in New Zealand and globally. Education relating to the health consequences of smoking tobacco is widespread and is accompanied by governmental policies to restrict the purchase and use, yet there are still too many individuals not embracing the opportunity to improve their health [9, 10]. A number of studies have been conducted to identify any perceived barriers deterring individuals from attempting cessation and furthermore investigating why some relapse. Several of these studies concluded a fear of weight gain was a common barrier for cessation and contributed to relapse [6, 11–14]. This information was the driver behind researchers initiating investigations into the effectiveness of pharmacological aids to attenuate weight gain post smoking cessation. The aim of this review is to assess the effectiveness of naltrexone combined with currently available smoking cessation medications to reduce post smoking cessation weight gain. The current literature will be reviewed with the objective being to investigate a possible solution that may remove a perceived barrier to smoking cessation and achieve the overall goal of a reduction in the number of smokers thus reducing the impact on individuals and health systems. A brief outline of the literature highlighting perceived barriers to smoking cessation and causes of relapse will be followed by an outline of the pharmacological aspects of naltrexone that support an investigation in to its use for weight control post smoking cessation. A summary of the current research obtained will be followed by a critical discussion on the findings and the conclusion will summarise these findings and identify any gaps in the research.

Despite global efforts to encourage smoking cessation and evidence of an overall decline in the number of smokers, 21 % of individuals aged over fifteen still smoke [1, 15]. Studies have revealed more than two thirds of current smokers wish to quit; however only half will attempt cessation and approximately two thirds of abstainers’ relapse in the first year [12, 16, 17]. This highlights a need for advancements in cessation methods to reach those for whom the current initiatives are not working [6, 12, 13]. Although research has revealed evidence that the majority of smokers are aware of the harm smoking causes [18], it also highlights a fear of weight gain as being a prevalent deterrent for attempting cessation, and a contributing factor to relapse with both these factors underpinned by the belief that smoking tobacco helps control weight [13, 19, 20].

With the introduction of tobacco, tobacco companies marketed cigarettes as a weight control product targeting women and this ideological belief appears to still be present in a number of smokers today [14, 21–23]. It is also apparent from the literature women allude to the fear of weight gain as being a barrier to smoking cessation more often than men and are three to four times more likely to relapse due to weight gain; however, it is acknowledged that some men do admit to believing smoking helps regulate their weight [12, 14, 22, 23]. Medical literature gives some substance to this notion of tobacco smoking physiologically regulating weight and impacting on eating behaviour. There is experimental evidence to suggest nicotine from tobacco smoking is associated with neuroadaptations that suppress reward driven eating and impact on resting metabolic rates whilst also increasing energy expenditure [13, 14, 19, 21, 23]. During smoking cessation and the subsequent withdrawal of nicotine, these anorectic effects are supressed and without action to control previously blunted eating behaviours weight gain is likely [14, 21, 24]. Studies have shown up to 80 % of smokers gain on average between two and five kilograms in the first year after cessation; however, some will gain in excess of ten kilograms and women are more likely than men to gain substantial weight [1, 21–23, 25]. It was also recognised the average number of cigarettes smoked daily had a direct correlation to the amount of post cessation weight gain, the more an individual smoked the more weight they tended to gain post cessation [1, 14, 21, 22, 24, 25]. In a prospective study performed in New Zealand it was noted that even though smokers who quit gained more weight than those who continued to smoke, in general the gain was no more than those of similar age, who had never smoked, gained over the same time [26]. Despite varying evidence that suggests cigarette smoking can be implicated in weight control, other evidence reveals a substantial number of current smokers are classed as obese [11, 14, 24, 27]. One must question which came first the obesity or the smoking and are these smokers trying to reduce the chance of further weight gain by smoking? Yu et al., (2014) [24] found that obese smokers were less likely to be prescribed smoking cessation medication and questioned whether this was due to the fear of further weight gain preventing them seeking help or whether health professionals fear of further weight gain stopped them prescribing. Furthermore, studies have shown any weight gain post smoking cessation can be attributed to an increase in the incidence of type two diabetes and contributes to an increase in the risk of hypertension by up to 30 % which surmounts to very high risk for those already obese [11, 13, 21, 22, 25]. In summary the literature supports the theory that post cessation weight gain is a warranted barrier to attempting cessation and increasing the risk of relapse especially in women. Therefore, offering a solution to decrease the risk of weight gain post cessation may encourage and help individuals still smoking to successfully and permanently quit. First it is important to ascertain if the currently available cessation medications have an effect on post cessation weight gain.

To date clinical trials testing various currently available smoking cessation medications capacity to attenuate post cessation weight gain alone have returned mixed results. The most commonly used cessation medications include nicotine replacement therapy in the form of patches, bupropion, and varenicline [10]. Yang et al., (2016) [28], found that although participants receiving bupropion appeared to gain less weight than participants using varenicline, once the results were adjusted for confounding factors they were deemed to be statistically insignificant. Alternately Schnoll et al., (2012) [20] found participants using nicotine patches for longer than the standard cessation period appeared to gain less weight than those using them for the standard time frame and adherence to patch use was greater in the longer term participants. However, a limitation of this study was that patch adherence was determined by self-reporting which may affect the validity of the results [29]. Bush et al., (2012) [22], trialled cognitive behavioural therapy as a combination with nicotine patches to attenuate post cessation weight gain with positive results; however, once again self-reporting and surveys were used to acquire results. The mixed results received from these studies warrant further investigation in to alternate therapies such as combination pharmacotherapies. Naltrexone combined with existing cessation medications has been touted as a possible pharmacological combination that may be successful in helping to achieve this [9, 17, 30–34].

### Naltrexone

Naltrexone is a semi synthetic opioid that acts as an antagonist at the μ receptors in the endogenous opioid system in the brain and is currently approved to treat opioid addiction and alcohol dependence [35, 36]. It has also recently been approved as an adjunct treatment to assist weight loss in morbidly obese individuals in the United States [34–37]. The endogenous opioid system has been implicated in hedonic eating behaviours and evidence suggests there is interaction between the opioid and nicotinic system, which is implicated in smoking addiction [17, 19, 31, 34, 38]. As mentioned previously post cessation weight gain can be partly attributed to hedonic eating behaviours that were formerly blunted by nicotine from tobacco smoking. Other sources of nicotine have also been proven to decrease hedonic eating in non-smokers which further supports the theory that withdrawing nicotine can increase the likelihood of hedonic eating behaviour leading to weight gain [19,]. Naltrexone has been proven to reduce eating behaviour synonymous with the endogenous opioid pathway by stopping the rewarding effects and increasing aversion to palatable foods (those high in fat and sugar) [19, 35–38]. Murray et al., (2014) [38], conducted a study to test the efficacy of naltrexone to blunt the desire for palatable food, and found participants administered naltrexone displayed a decreased response in reward stimuli to palatable foods and an increased aversion to other foods when compared with placebo. A systematic review and meta -analysis investigating the use of opioid antagonists including naltrexone as a monotherapy for smoking cessation completed by David et al., (2014) [39], concluded that there was no evidence of any benefit of naltrexone on its own as a therapy to aid smoking cessation. However, there is evidence that supports naltrexone being an effective tool to halt eating behaviour driven by effects of the opioid pathway which are perceived to be enhanced by smoking cessation. The possibility that it could be effective as a pharmacological combination with approved smoking cessation medications to reduce post smoking cessation weight gain warranted further investigation and a number of studies have been conducted.

### Method

To procure the current relevant literature for review a thorough search of the Auckland University of Technology library including the databases, CINAHL, EBSCO Health, Google Scholar, Medline, ProQuest, Science Direct and Scopus was performed. Each source was individually searched using the keywords and phrases “naltrexone”, “smoking cessation”, “weight reduction”, “opioid antagonists”, “barriers to smoking cessation”, “obesity and smoking cessation”, and “tobacco control strategies”. To access studies directly relating to the use of naltrexone for post smoking cessation weight reduction a combination of these phrases was used: “naltrexone” and “smoking cessation” and “weight reduction”. Furthermore, to ensure the results returned were current and from credible sources further parameters were set. Primarily studies that were published in peer reviewed journals and had been conducted within the past ten years were included. With advancements in pharmacotherapy to aid smoking cessation the authors deemed it necessary to consider research applicable to currently available smoking cessation medications hence any studies pre-dating 2006 were excluded as were studies using monotherapy: however, these studies were utilised for background information.

## Discussion

### Effectiveness of naltrexone to attenuate post smoking cessation

Several studies have tested the effectiveness of naltrexone to attenuate post smoking cessation weight gain. Of the seven studies found, five were double blinded randomised placebo controlled trials (RCT), one was an open label study with a control group, and one was an open label study with no control group. The studies all measured outcomes within the fifty-two weeks immediately post cessation which is in line with evidence suggesting the greatest weight gain usually occurs in the first year post cessation [21]. Five out of seven studies returned positive results suggesting a naltrexone combination could be effective in reducing post smoking cessation weight gain while two found results that were statistically insignificant [9, 10, 17, 32, 33, 40, 41]. Four of the studies returning positive results used a combination of nicotine patches and naltrexone and the remaining one used bupropion combined with the naltrexone [10, 32, 33, 41]. One of the studies that revealed statistically insignificant results used the nicotine patch combination and the other the bupropion [9, 17]. Of note, there appears to be no current studies trialling a combination including the other most commonly prescribed smoking cessation medication, varenicline. In previous studies, the two medications that were trialled, nicotine patches and bupropion, although results were deemed statistically insignificant appeared to have more positive effect on reducing post cessation weight gain as a monotherapy than varenicline [28]. However, varenicline has returned the best long term abstinence rates and is generally considered the most effective smoking cessation medication [10, 28]. One must question why a combination of naltrexone and varenicline appears to have not been tested thus far when existing evidence suggests it may produce better abstinence rates. Furthermore, having reviewed the pharmacological datasheet on varenicline there does not appear to be any identified interactions between naltrexone and varenicline that could prevent a combination being prescribed [42]. Nevertheless, nicotine patches with naltrexone were the most common combination used in the studies, this could possibly be due to less reported side effects from nicotine patch use as a monotherapy compared with bupropion and varenicline [43]. It is important to acknowledge all of the studies included pertaining to the effectiveness of naltrexone combined with smoking cessation medication to attenuate post cessation weight gain were conducted in the United States of America and several researchers were involved in more than one of the studies. Upon analysis overall the available studies returned varying results. A number of common themes including the role of hedonic eating in post cessation weight gain and treatment implications, abstinence rates, and the tolerability of naltrexone, were apparent.

### Sex specific effectiveness of naltrexone

King et al., (2012) [32] and King et al., (2013a) [33] initiated studies where the aim was to assess the effectiveness and ascertain if there were any sex specific results when using a combination of naltrexone, nicotine patches, and cognitive behavioural treatment (CBT) to attenuate post cessation weight gain. Both the studies found that the women in the studies showed significantly reduced weight gain compared with the placebo group whereas the men did not. However, the women in the placebo group had greater weight gain than the men in the placebo group. A possible explanation for these results as mentioned previously could be due to the fact that some women have been shown to have lower cognitive control of brain responses to food stimuli and are somewhat predisposed to hedonic eating behaviour which during smoking is blunted by the effects of nicotine. Once nicotine is withdrawn and hedonic behaviours are amplified weight gain is inevitable if control is not regained hence those taking naltrexone gained less weight [31]. On the other hand, the results of a study undertaken by Toll et al., (2010) [9], investigating the effects of naltrexone combined with nicotine patches and CBT in highly weight concerned smokers returned negative results and may further support the notion that the lack of cognitive control apparent in some women may be implicated in post cessation weight gain. Participants included in this study were recruited according to their score on a weight concern scale and as women scored the highest the study included more women than men. The results showed that there was no significant difference in weight gain between the treatment group (receiving naltrexone) and the placebo group. A suggested reason for this was participants already had cognitive control over their eating and although advised not to, may have dieted throughout treatment [9]. Dieting is regarded as counterproductive during smoking cessation as it is suggested those who restrain their eating habits because of fear of weight gain are more likely to relapse due to a sub conscious reinforcement of smoking as a weight control mechanism [9]. This may offer an explanation for the extraordinarily high dropout rate from this particular study [9, 19]. The evidence from these studies suggest that to some extent naltrexone may be more effective for women than men due to commonly perceived differences in physiological driven eating behaviours [9].

### Use of naltrexone in obese patients

Wilcox et al., (2010) [10], tested the use of a naltrexone with bupropion combination and behavioural therapies in already overweight and obese smokers. On completion of the study it was found there was no significant change in the weight measurements of participants when compared with their baseline measures. Existing evidence supports the perception that overweight and obese individuals may have low conscious control over their eating habits and are also prone to display hedonic eating behaviour [11, 19, 34, 35]. The study results support the hypothesis that naltrexone may curb this behaviour leading to reduced weight gain post smoking cessation [10]. However, there was no control group in this study so there is no evidence to prove the effects were not produced by the bupropion or behavioural therapy. In fact, efficacy studies indicate that specialised behavioural therapy has been successful in suppressing weight gain over the short term during smoking cessation [22]. Whilst King et al., (2012) [32] also included individuals in their research that were classed as overweight and obese the results were not stratified by body mass index. It is hard to determine if the positive results were due to the inclusion of obese and overweight participants. However, if compared with the Toll et al., (2010) [9], study which only included highly weight concerned smokers who fitted in the normal weight category and returned negative results, assumptions may be made that suggest the results could have been positive due to the inclusion of overweight and obese participants who were more likely to exhibit hedonic eating behaviour. There is some suggestion that naltrexone may be effective in overweight or obese individuals to attenuate post cessation weight gain. Even though the aim of this review is to assess the effectiveness of naltrexone regarding weight gain it is also important to consider the effect of naltrexone on abstinence rates. It would be counterproductive to offer a weight reduction pharmacotherapy that negatively affects abstinence rates.

### Effect of naltrexone on cessation rates

There are mixed results reported in the literature regarding the possible effect of naltrexone on cessation rates. In two studies that extrapolated sex specific results, the men from the treatment group returned better abstinence rates than the placebo group, and the women in the treatment group [32, 33]. In contrast Toll et al., (2010) [9], found in a similar RCT combining nicotine patches and CBT with naltrexone that abstinence rates were greater in the placebo group than the treatment group. However, when considering the results from the sex stratified studies the ratio of men to women in the Toll et al., 2010 [9] study placebo group may have impacted on the results and furthermore according to Walker et al., (2016) [15], evidence shows that men are more likely than women to achieve and maintain abstinence. Whilst King et al., (2013b) [17], Wilcox et al., (2010) [10] and Toll et al., (2010) [9] found naltrexone appeared to reduce the urge to smoke and the number of cigarettes smoked pre cessation, this could have been attributed to the cessation medications, nicotine patches and bupropion, as during an investigation by Rohsenow et al., (2007) [44] in to the effects of naltrexone on smoking cessation it was found naltrexone did not reduce the urge to smoke after ten hours of nicotine deprivation. In a small open label study conducted by Toll et al., (2008) [41] with the aim of testing what effect a combination of bupropion and naltrexone may have on weight gain post cessation, the participants receiving bupropion monotherapy maintained a better rate of abstinence post treatment than those taking the naltrexone combination. However, due the extremely small number of participants in this study the results cannot be deemed as indicative of expected results in a larger population [29]. Whilst all previously mentioned studies trialled combinations that included either 25 mg or 50 mg doses of naltrexone O’Malley et al., (2006) [40], tested the effects of a combination including a 100 mg dose of naltrexone, nicotine patch and CBT and found that those receiving the highest dose of naltrexone, 100 mg, returned better abstinence rates during the treatment period than those in the lower dose groups and those receiving placebo but these results were not observed in a post treatment follow up. It appears that whilst naltrexone may have shown better results in abstinence in men in two of the studies overall the placebo groups appeared to sustain a higher rate of abstinence. An important factor that these studies appeared to neglect was the number of previous quit attempts participants had made. Although several excluded those individuals who had made recent quit attempts, evidence from large study by Chaiton et al., (2016) [45] highlights it may take a smoker on average thirty attempts to quit before being successful. This raises questions on the validity of results correlating abstinence to type of therapy used unless quit attempts have been included as a possible confounder [29]. Tolerability and adherence to therapies can also effect overall results but were well documented during the included studies analyses.

### Side effects of naltrexone use in smoking cessation therapy

The most common side effects attributed to naltrexone throughout the studies were nausea and dizziness; however, these were generally reported to be mild and on the whole were said not to have had an effect on overall adherence rates [9, 10, 17, 32, 33, 41]. It is important to note that nausea and dizziness are also common side effects of nicotine patches and even though there appeared to be less incidence in the placebo groups there is no definitive evidence to surmise which pharmacotherapy caused these effects as every individual reacts differently and more in the treatment group may have reacted to the nicotine patch [20, 46]. There was one exception to the generally acceptable adherence rates, O’Malley et al., (2006) [40] found that participants receiving a naltrexone dose of 100 mg did not show as greater compliance rates as those receiving the lower doses of 25 mg or 50 mg due to the persistence of unpleasant side effects. At the 100 mg dose there were also four cases of increased liver function values deemed to be outside the safe threshold and once naltrexone was stopped they returned to normal [40]. Liver function tests from the lower dose participants in the O’Malley et al., (2006) [40] study and the Toll et al., (2010) [9] study were all normal and within the safe range throughout the duration of treatment. Furthermore, in the O’Malley et al., (2006) [40] study participants in the 25 mg treatment group reported side effects that were no different to those experienced by participants in the placebo group. Overall evidence suggests that 25 mg and 50 mg naltrexone were well received with few side effects and generally good tolerability.

## Conclusions

In conclusion the evidence suggests that naltrexone combined with existing approved smoking cessation medications may be an effective pharmacotherapy to attenuate post smoking cessation weight gain in individuals whom are more likely to display hedonic eating behaviours, for example some women and those individuals who are already overweight or obese. This is promising as the literature reiterates that women and obese individuals are more likely to not attempt cessation due to fear of weight gain and furthermore women are three to four times more likely than men to relapse because of weight gain. A noticeable gap in the research pertained to the apparent exclusion in trials of a naltrexone combination with varenicline which is deemed to be the cessation medication that produces the best long term abstinence rates. Further research trialling varenicline and stratifying results by gender and weight status is recommended to find the most effective combination. Whilst abstinence rates did not appear to be significantly affected by the use of naltrexone and although men appeared to maintain more favourable abstinence results whilst receiving treatment with a naltrexone combination, without further gender specific research there is not enough evidence to draw conclusions other than naltrexone does not appear to have any negative effects on abstinence rates. Overall the combinations including the 25 mg and 50 mg naltrexone components were well tolerated by study participants and adherence rates were satisfactory. With smoking still causing the greatest number of preventable deaths globally and weight gain being touted as a significant barrier to attempting cessation whilst also contributing to relapse, the evidence suggests the use of naltrexone to attenuate post cessation weight gain may provide a solution. In a New Zealand context although regarded as leaders in tobacco control there are still sectors of society that are grossly overrepresented in smoking statistics and need to be reached. Māori women are one such group and with the positive results seen in the trials for women a naltrexone combination may help reduce these numbers by removing an identified barrier to cessation. With further research to find the optimal combination naltrexone could provide the solution to removing this barrier thus helping increase global smoking cessation rates and relieving unnecessary burden on individuals and healthcare systems. It may provide the impetus New Zealand needs to reach the goal of Smokefree 2025.

## Abbreviations

CBT: Cognitive behavioural treatment
RCT: Placebo controlled trials
